# Stellate Ganglion Block for Post-traumatic Stress Disorder: A Comprehensive Review of Evidence, Technique Considerations and Symptom Outcomes in Military and Non-Military Patients

**DOI:** 10.1007/s11920-026-01666-4

**Published:** 2026-03-18

**Authors:** Thomas Bielawiec, Brittany Melvin, Bhuvaneswari Sandeep Ram, Magdalena Anitescu

**Affiliations:** Department of Anesthesiology and Critical Care Section of Pain Medicine, University of Chicago Medical Center, Chicago, IL USA

## Abstract

**Purpose of Review:**

Post-traumatic stress disorder (PTSD) is a psychiatric disorder which is defined by four symptom clusters of intrusive re-experiencing, avoidance, negative mood/cognitive changes, and hyperarousal. This pathway is mediated by a dysregulation in the amygdala-hippocampus-medial prefrontal cortex circuit resulting in an abnormal threat processing and other symptoms. Stellate ganglion blocks (SGB) are largely supported in a review of the current literature. The objective of this review is to synthesize available evidence and extrapolate findings to identify patient and symptom profiles most likely to benefit, as well as technique variations that may optimize clinical outcomes.

**Recent Findings:**

Bilateral SGBs or blocks in conjunction with a superior cervical ganglion block (SCGB) have been studied and appear safe and effective but may not necessarily offer superior efficacy or durability of response, although may be considered in patients that fail a right sided block.

**Summary:**

In observations across studies, hyperarousal symptoms tended to benefit most and re-experiencing the least. The effects are positive in both mild-moderate and severe cases, but severe cases may benefit more. The block has been shown to be effective in both military and non-military patients across all trauma types. The practitioner should perform the block using ultrasound or fluoroscopy based on their preference, and perhaps at least 6 mL of local anesthetic should be used. The block can be performed bilaterally (after 24 h to avoid bilateral recurrent laryngeal block) or in conjunction with a superior cervical ganglion block (SCGB), which has shown to be effective and safe, however not necessarily more effective than a single right sided block. There may be benefit in patients that fail a right-side block. The effects of a SGB on PTSD may last up to 3–6 months and can be repeated with efficacy. Ideally, PTSD should be treated in a multimodal approach using concurrent therapy and pharmacologic treatment. Further high-quality studies are needed to provide further data and evidence to support these observations.

## PTSD Background

PTSD is a psychiatric condition that develops after exposure to, or the threat of, traumatic events such as combat, interpersonal violence, severe accidents, or life-threatening illness. It is associated with chronic anxiety, depression, and significant functional impairment, leading to a marked reduction in quality of life [1]. Given its long historical trajectory and the continued refinement of its diagnostic framework, current research increasingly emphasizes optimizing treatment strategies that address both the neurobiological underpinnings and the complex psychological sequelae of PTSD.

### Prevalence

In the general U.S. population, the lifetime prevalence of PTSD is approximately 7.8%, with women nearly twice as likely to be affected as men [2–4]. Rates are consistently higher among military veterans due to combat-related trauma. The highest prevalence has been reported in Vietnam War veterans, with lifetime estimates approaching 30% and persistent symptoms in up to 15% [2, 5, 6]. Similarly, nearly 28% of Iran-Iraq War veterans met diagnostic criteria [7]. In contrast, prevalence in Gulf War and more recent conflict veterans – including those from Operations Enduring Freedom and Iraqi Freedom (OEF/OIF)- ranges from 5 to 13% [8–10].

These variations suggest that PTSD burden varies by conflict, with earlier wars such as Vietnam and Iran–Iraq carrying the highest risk. Contributing factors include combat intensity, delayed recognition of PTSD as a clinical diagnosis, and variable access to psychiatric care [5, 11]. More recent conflicts show lower prevalence, likely reflecting advances in medical support, systematic screening, and earlier intervention [9, 10]. Overall, PTSD remains markedly more common in veterans than in civilians and is frequently complicated by comorbid psychiatric disorders and elevated suicide risk.

### Neuro-hormonal and Neurotransmitter Dysregulation in the Pathophysiology of PTSD

PTSD is characterized by neuro-hormonal dysregulation and structural alterations in the hippocampus, a region critical for memory, emotion, and sympathetic nervous system (SNS) regulation [12–14]. Dysregulated hippocampal-Papez circuit signaling contributes to exaggerated hypothalamic activity, over-activation of the hypothalamic-pituitary-adrenal (HPA) axis, and heightened SNS output [12, 14]. Chronic stress with sustained elevations in corticotropin-releasing factor (CRF) drives cortisol dysregulation, hippocampal atrophy, and impaired emotional regulation [15–17].

At the neurotransmitter level, PTSD is associated with elevated norepinephrine (hyperarousal, intrusive memories), reduced serotonin (anxiety, irritability), decreased GABA (loss of inhibitory control), increased glutamate and CRF (excitatory stress drive), and reduced neuropeptide Y (NPY) [12, 14, 17, 18]. A summary of these mediators and their dysregulation is provided in Table 1.

**Table 1:** Key Neuro-hormonal and neurotransmitter imbalances in PTSD

System or Neurotransmitter	Imbalance in PTSD	Clinical Effects
HPA Axis (cortisol)	Decreased	Hyperarousal, impaired stress adaptation, impaired stress adaptation
Serotonin (5-HT)	Decreased	Depressed mood, anxiety, sleep disturbance
Dopamine	Decreased	Impaired award processing, emotional dysregulation
GABA	Decreased	Anxiety, impaired fear inhibition, hyperarousal
Glutamate	Elevated	Excitotoxicity, impaired fear extinction.
Corticotropin-releasing hormone (CRH)	Elevated	Heightened stress response, anxiety, sleep disturbance
Norepinephrine	Elevated	Hypervigilance, sleep disturbance, intrusive symptoms
Opioid System	Dysregulated	Altered pain perception, emotional numbing

### Clinical Picture and Diagnostic Tools for PTSD

Currently, the DSM-5 defines PTSD by four symptom clusters: intrusive re-experiencing, avoidance, negative alterations in cognition and mood, and heightened arousal/reactivity [1, 19]. Intrusive symptoms include recurrent memories, nightmares, and flashbacks. Avoidance reflects efforts to evade trauma-related reminders. Cognitive and mood alterations encompass guilt, persistent negative affect, and diminished interest in activities. Arousal symptoms include irritability, hypervigilance, impaired concentration, and sleep disturbance. Diagnostic criteria require that symptoms persist for more than one month, cause significant impairment, and not be attributable to substance use or other medical conditions [1]. The International Classification of Diseases 11th Revision (ICD-11) definition only requires three core symptom clusters which are reexperiencing in the present, avoidance, and persistent perceptions of heightened current threat [20]. It also has a separate diagnosis of complex PTSD which includes three additional clusters: affect dysregulation, negative self-concept, and interpersonal difficulties.

**Fig. 1:**
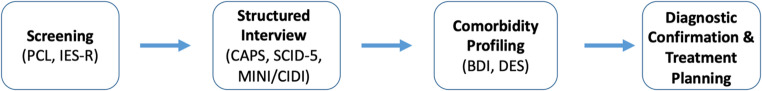
Stepped diagnostic approach to PTSD. Screening tools (PCL, IES-R) are followed by structured interviews (CAPS, SCID-5, MINI/CIDI) and comorbidity assessments (BDI, DES) to ensure accurate diagnosis and guide treatment planning

Diagnosis is based on clinical evaluation, supported by validated instruments (Table 2). The Clinician-Administered PTSD Scale (CAPS) is considered the gold-standard interview, providing a direct assessment of the frequency and severity of DSM-based symptoms [21]. Self-report measures, including the PTSD Checklist (PCL) and the Impact of Event Scale-Revised (IES-R), are widely used to quantify symptoms burden [22, 23]. Forms such as PCL-M and PCL-C exist that are specifically tailored to military or civilian populations. Structured diagnostic interviews such as the Structured Clinical Interview for DSM-5 (SCID-5), the Mini International Neuropsychiatric Interview (MINI), and the Composite International Diagnostic Interview (CIDI), facilitate diagnosis in both clinical and research contexts [24, 25]. Additional tools, such as the Beck Depression Inventory (BDI) and Dissociative Experiences Scale (DES), are often employed to identify common comorbidities, including depression and dissociation [26]. Because no single test serves as a definitive diagnostic standard, a comprehensive psychiatric evaluation remains essential for confirming diagnosis and guiding treatment planning (Fig. 1).

**Table 2:** PTSD diagnostic instruments

Diagnostic Instruments	Primary Purpose
CAPS (Clinician-Administered PTSD Scale)	Gold standard structured interview; assesses symptom frequency/severity
PTSD Checklist (PCL)	Self-report; screens and monitors symptom burden
Impact of Event Scale-Revised (IES-R)	Self-report; measures trauma-related distress
Structured Clinical Interview for DSM-5 (SCID-5)	Structured interview; diagnostic confirmation and comorbidities
Mini International Neuropsychiatric Interview (MINI) / Composite International Diagnostic Interview (CIDI)	Brief structured interviews; efficient diagnosis in clinical/research settings
Beck Depression Inventory (BDI) / Dissociative Experiences Scale (DES)	Assess comorbid depression and dissociative symptoms

### Treatment of PTSD

The treatment of PTSD relies on a combination of psychotherapy and pharmacological interventions, with trauma-focused therapies serving as the foundation of care. Pharmacological options such as serotonin reuptake inhibitors (SSRIs; e.g. sertraline, paroxetine) and serotonin-norepinephrine reuptake inhibitors (SNRIs; e.g. duloxetine, venlafaxine) remain first-line treatments. However, response rates are typically below 50%, and adverse effects—including sexual dysfunction, weight gain, and sleep disturbance—often limit adherence [27, 28]. Prazosin has demonstrated efficacy for trauma-related nightmares, but its benefits are largely limited to sleep-related symptoms. These limitations emphasize that pharmacotherapy is most effective as an adjunct to psychotherapy rather than as a stand-alone treatment.

Psychotherapy provides the most durable outcomes. Prolonged Exposure Therapy (PE), Cognitive Processing Therapy (CPT), and Eye Movement Desensitization and Reprocessing (EMDR) target avoidance, maladaptive cognitions, and traumatic memory integration, consistently yielding substantial and sustained improvements [28–30]. Despite strong empirical support, clinician uptake remains limited due to concerns about patient destabilization and discomfort with structured, manualized protocols [31, 32].

Emerging approaches reflect a growing emphasis on personalization and accessibility. Virtual Reality Exposure Therapy (VRET) delivers immersive trauma-related environments, while mindfulness-based interventions improve emotional regulation and interoceptive awareness [27]. Psychedelic-assisted therapies, particularly MDMA with psychotherapy, have shown promise in treatment-resistant PTSD but remain experimental and subject to strict regulation. Other innovative modalities, such as Visual Schema Displacement Therapy (VSDT), artificial intelligence-based tools, and 3D simulations, remain in early stages of evaluation.

Overall, the management of PTSD has shifted from reliance on pharmacological monotherapy toward integrated, multimodal strategies. Nonetheless, persistent gaps in efficacy and implementation underscore the need for novel interventions, including SGBs.

### Stellate Ganglion Block (SGB)

Stellate ganglion blocks (SGBs) have been investigated as an interventional treatment option for PTSD. SGB is a minimally invasive procedure in which local anesthetic is injected at the C6 or C7 vertebral level to target the stellate ganglion which is composed of the inferior cervical ganglion and the first thoracic ganglion fusion, thought to be found in approximately 80% of the population [33]. Blockage is typically done at the C6 level as it has a greater safety profile due to the vertebral artery being protected in the transverse foramen and farther away from the brachial plexus and lung pleura. At this level, it is more accurately described as a cervical sympathetic block since only traversing sympathetic fibers or middle cervical ganglia are found here. The stellate ganglion carries both preganglionic fibers and postganglionic fibers that provide sympathetic innervation of the upper limbs and head and play a role in driving the nervous system’s “fight or flight” response. In attempt to achieve a more complete cervical sympathetic blockade, some clinicians additionally target the cervical sympathetic ganglia at C3 or C4, which aims to address potential anatomy variability in sympathetic innervation given that the stellate ganglion is a fusion of nerves. However, this involves a second injection which may increase the risk of overall injury to neurovascular structures and involves higher local anesthetic volumes. The precise mechanism of the block has been a topic of investigation, however newer theories suggest that the block may reduce the amount of nerve growth factor and neurite outgrowth, causing a decrease in norepinephrine and sympathetic activity [34, 35]. Classically, the block has been performed on the right side because of the neuroanatomical association of the right central autonomic network and maintenance of chronic sympathetic response [36]. This block has been utilized in pain conditions in the head, upper limbs, refractory angina and cardiac arrhythmias that occur as a product of abnormal connections between the sympathetic and sensory nervous systems. Although generally safe and well tolerated, possible complications have included neurovascular injury, pneumothorax, thyroid injury, esophageal and tracheal puncture, Horner’s syndrome (although expected) and infections [37].

### Methods

**Fig. 2:**
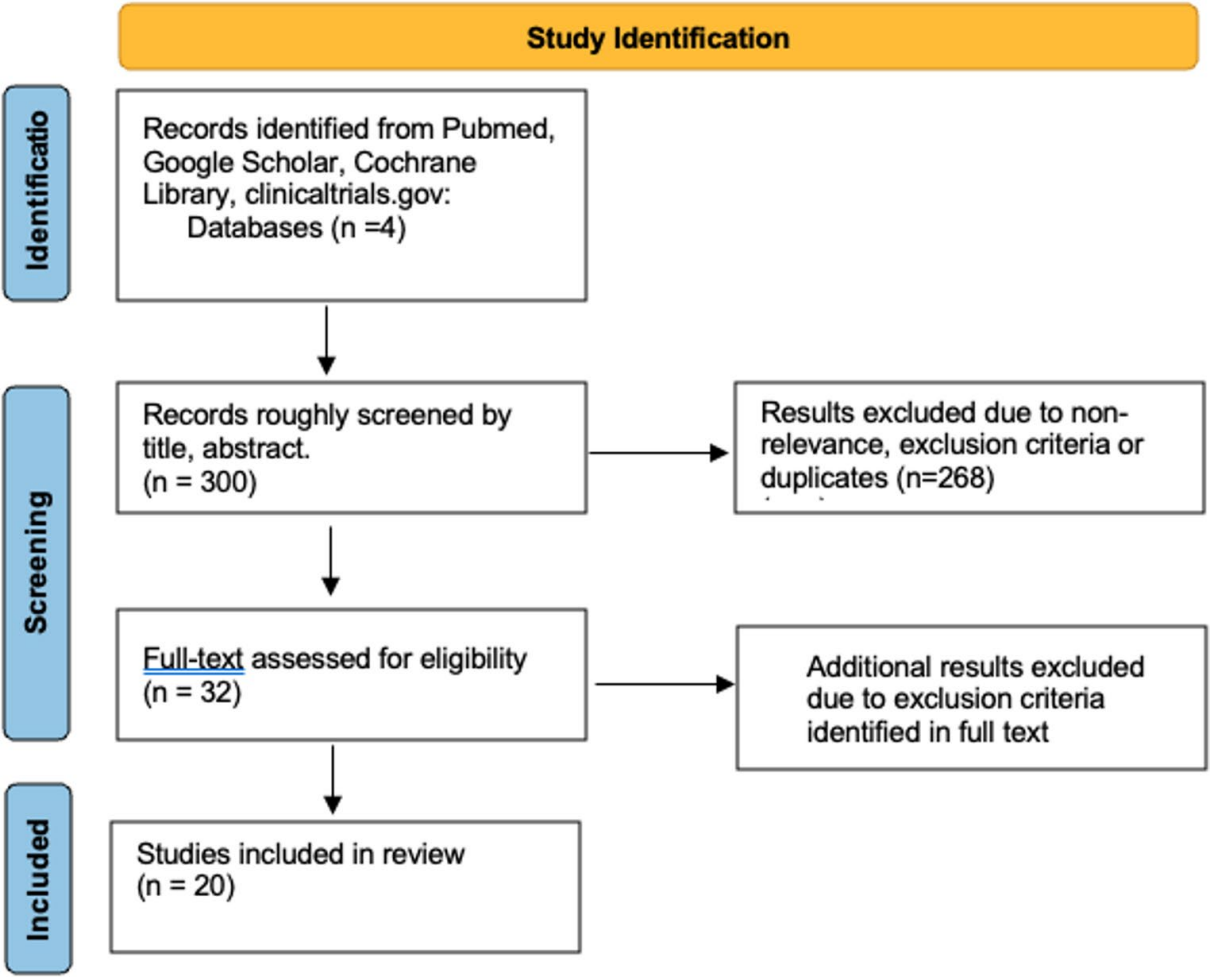
Study Identification. Template adapted from PRISMA [44]

A comprehensive search was conducted to identify literature that looked at the effectiveness of SGBs in patients diagnosed with PTSD. PubMed, Google Scholar, Cochrane Library and clinicaltrials.gov were used as primary databases. Clinicaltrials.gov was reviewed for in-process studies. All randomized control studies, case control studies, retrospective cohort studies, case series with a minimum of 3 patients and meta-analysis studies in English were included with adult patients formally diagnosed with PTSD (via DSM-4 or DSM-5 criteria) with at least 1 follow up post injection that included a validated scale such as PCL or CAPS. Systematic reviews were reviewed for any additional omitted studies. Studies including case reports with 1–2 patients, expert opinions, narrative reviews, analyzing pediatric patients or patients with PTSD due to organic brain disorders and those without validated measures to assess PTSD symptom improvement were excluded. The patient and interventional procedure details such as trauma type, demographics, laterality, single or multiple injections, block repetition, inclusion of superior cervical ganglion block, type of local, use of fluoroscopy vs. ultrasound, concurrent treatment therapies, follow up intervals and outcomes were noted, focusing on symptom clusters where available. A search of PubMed using the terms “PTSD” and “stellate ganglion block” between the year 2000 and October 2025 resulted in 63 results, from which 18 met inclusion criteria. A search on Google Scholar with the same search terms and after review of the first 200 out of 1700 + results yielded 2 additional included studies. There were no additional studies found reviewing 18 Cochrane Library results. Clinicaltrials.gov yielded 16 results of which none had data to be included. 4 actively ongoing randomized controlled trials were identified. No meta-analysis was identified. Keywords such as “cervical sympathetic block” and “superior cervical ganglion block” were not included so that the review can focus on clinically oriented studies related to stellate ganglion blocks in PTSD, however this methodological approach risks missing studies primarily focused on these interventions or worded under synonymous terms. Study identification is summarized in Fig. 2.

## Results/Discussion

The first reports of SGBs being used in mental disorders dates to 1947 where the block was observed to have positive effects on depression [39]. In 1990, the stellate ganglion block was first described in its use in PTSD in conjunction with pharmacologic treatment and therapy [40]. Interest in the topic began to pick up in the 2000s during which all the reviewed literature was published (Table 3).

**Table 3:**
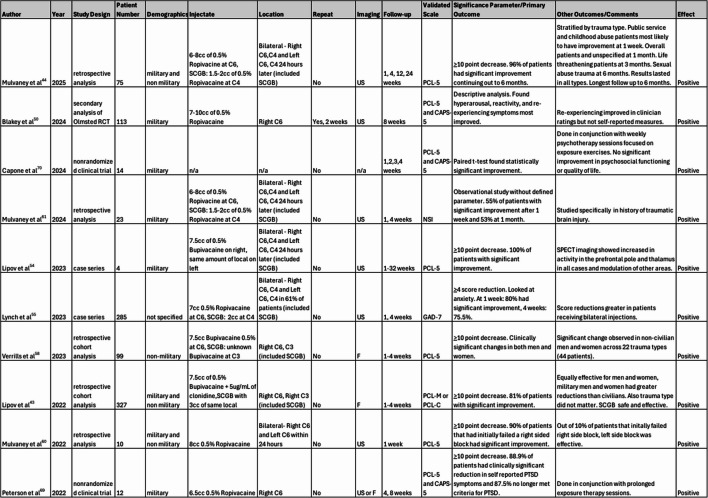

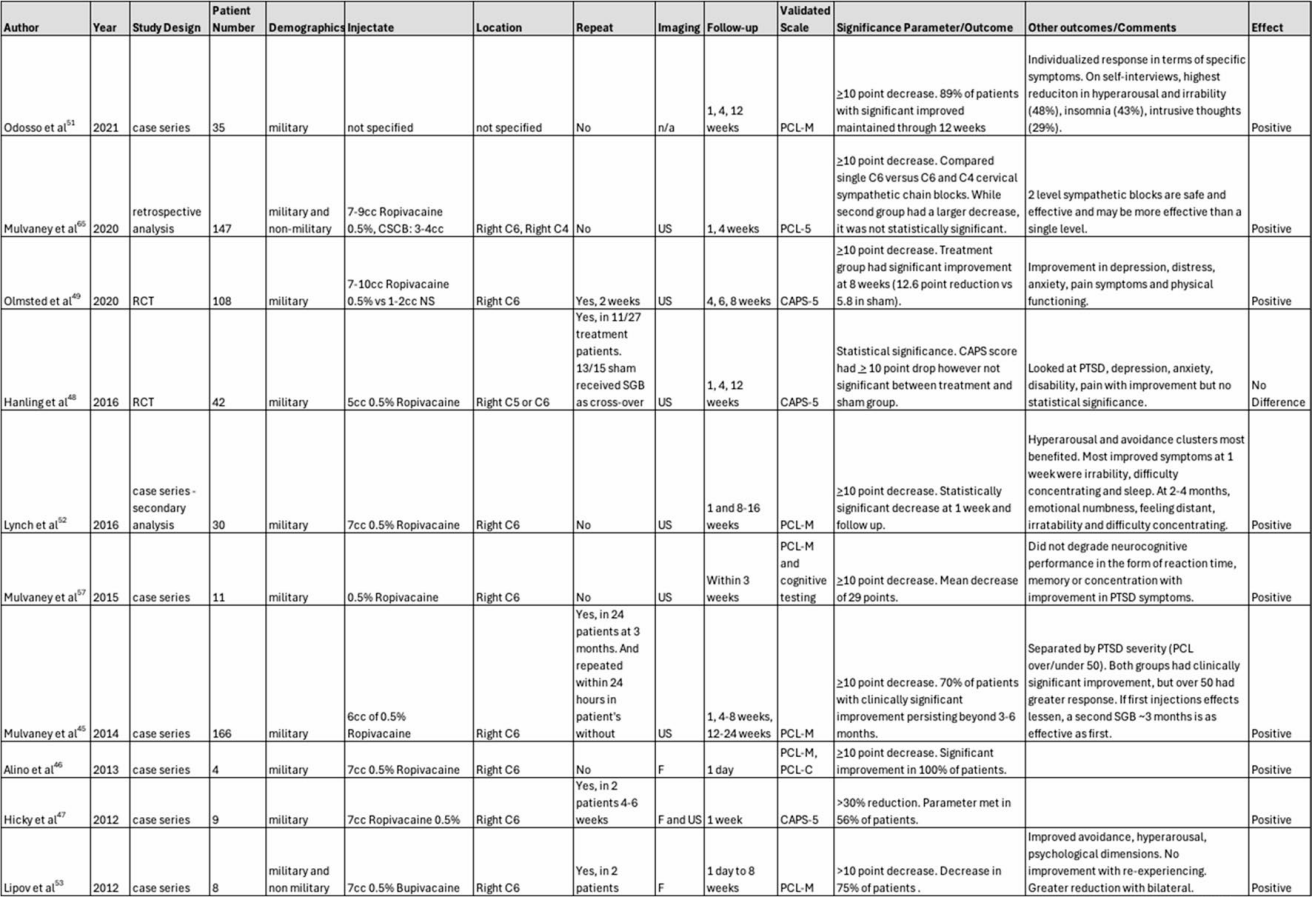
Summary of included studies including technique details, follow up and outcomes. SCGB: superior cervical ganglion block, PCL-5: PTSD Checklist for DSM-5, PCL-M: PTSD Checklist for Military, PCL-C: PTSD Checklist for Civilian, RCT: randomized controlled trial, NS: normal saline, US: ultrasound, F: fluoroscopy, CAPS-5: Clinician Administered PTSD Scale for DSM-5, NSI: neurobehavioral symptom inventory, GAD-7: General Anxiety Disorder 7

Overall, most of the literature obtained for this review appears to support the clinical efficacy of SGB in PTSD patients, although most of the data comes from retrospective analysis and case series, with one RCT supporting this finding and one providing conflicting results. Most of these studies used PCL (PCL-M, PCL-C, PCL-5) or CAPS-5 scales to assess improvement in PTSD symptoms, using 10 points as a clinically significant improvement [41]. A 5–10-point decrease also represents a reliable change not due to chance. For PCL-5, a score of 31–33 offers a provisional diagnosis for PTSD [42]. Lipov et al. analyzed the largest retrospective cohort analysis involving 327 military and non-military patients, in which 81% of patients experienced significant improvement [43]. In other large (at least 75 patients), non-RCT studies using PCL, at least 70% of patients had clinically significant PCL improvement, with one study showing up to 96% [44, 45]. In smaller case series, the clinically significant decrease was observed in a range from 50 to 100% of patients [46, 47].

### Randomized Controlled Trials

While many of the case reports/series and retrospective studies showed positive results, the evidence in randomized controlled trials has been conflicting, however, only two randomized control trials have been performed to date.

In 2016, Hanling et al. published the results of the first randomized controlled trial of the efficacy of right sided SGB in PTSD which included 42 patients to receive SGB (*n* = 27) or sham injection (*n* = 15) [48]. The primary outcome measure was CAPS and other outcome measures included self-report scales of PTSD, depression, anxiety and disability. The outcomes were assessed pre-procedure, post-procedure at 1 week, 1 month and 3 months. The authors allowed 2:1 active versus sham with a crossover protocol based on the positive results reported in the literature thus far. Results indicated that observed PTSD symptoms (CAPS) improved in participants in both the active and sham groups and other secondary outcomes except pain. Cross-over participants did not show any difference between sham and SGB. The authors concluded that SGB did not offer any benefit in PTSD over placebo. They also reported a modest improvement in patients who received the second block in 29 patients (*P* < 0.05).

This was followed by a 2020 multicenter randomized trial by Olmsted et al. in which they used a 2:1 right sided SGB-Sham block in 113 patients with SGB (*n* = 74) and sham (*n* = 39) as 2 blocks administered in 2 weeks [49]. The primary outcome was CAPS measured baseline to 8 weeks. Results reported included an adjusted mean total symptom severity score change was − 12.6 points (95%CI, − 15.5 to − 9.7 points) for the group receiving SGB treatments, compared with − 6.1points (95%CI, − 9.8 to − 2.3 points) for those receiving sham treatment (*P* = 0.01) reporting effectiveness of reducing symptom severity in 8 weeks.

Conclusions between the two trials may have differed due to methodological differences. The sham procedure design in the Hanling trial injected saline near the stellate ganglion, which may have produced a therapeutic effect through compression of the ganglion alone and may explain improvement that was not statistically significant. The injectate in the Hanling trial was also 5 cc, which was less than what had typically been used previously. The Olmsted trial injected medication subcutaneously for the sham group. The Olmsted trial investigated a highly selected patient population (stable medication regimen, no traumatic brain injury, and overall low-to-moderate PTSD severity). The Hanling trial had broader inclusion criteria and enrolled a smaller patient population with follow ups limited to one week and one month. Olmsted also only followed patient out to two months. Interestingly, Hanling noted that participants receiving a second SGB showed greater improvement than after the first treatment, which may suggest potential dose-response effects. These second treatments and crossovers complicate interpretation of the primary conclusion. Secondary gain could not be eliminated as most of these patients were transitioning out of the military and undergoing medical disability evaluations, the placebo group was overweighed with 15 sham versus 27 SGB patients. For both trials, it is difficult to control for the Horner’s syndrome that can be seen with SGBs that may inform patients and assessors of sham versus treatment groups.

In 2024, Blakey et al. published the results of the secondary exploratory analysis of the Olmsted study where they confirmed the diagnosis and conducted logistic regression models followed by ad hoc exploratory analysis of each symptom cluster with the objective to identify which cluster of symptoms responds well to SGB. For CAPS-5, the largest differences in the proportion of service members who remitted from cluster level clinical threshold status were observed in the reexperiencing cluster (27.1% for SGB vs. 13.2% for sham) and the arousal and reactivity cluster (18.6% for SGB vs. 5.3% for sham). For PCL-5, the largest difference in the proportion of service members who remitted from cluster-level clinical threshold status was observed in the arousal and reactivity cluster (32.4% for SGB vs. 10.8% for sham) [50].

### Improvement in Symptom Clusters

While most of the evidence supports SGB in overall PTSD, of interest is whether any specific symptoms improve more than others. In one secondary descriptive analysis of a RCT involving 113 patients, hyperarousal, reactivity and re-experiencing symptoms were the most improved, although re-experiencing was per clinician ratings and not patient reported measures [49, 50]. In three additional smaller case series that looked at symptom clusters, the hyperarousal and avoidance clusters had the most benefit, which included symptoms such as irritability, outbursts, avoiding thoughts and feelings and emotional numbing [51–53]. Findings that SGBs in PTSD patients result in increased activity in the prefrontal pole and thalamus on SPECT imaging would be supported by these symptom improvements [54].

When compiling observations across all the studies, hyperarousal symptoms consistently showed the most benefit, while re-experiencing, which includes symptoms like intrusive thoughts, flashbacks and nightmares, had the least or no improvement for patients.

In terms of effects on anxiety and depression, one case series of 285 PTSD patients specifically looked at improvements in anxiety after SGB using the General Anxiety Disorder Scale (GAD-7) and found that there was a clinically meaningful improvement for at least a month [55]. This was a finding that was also observed in the Olmsted et al. RCT along with improvements in depression, anxiety, pain symptoms and physical functioning [49]. However, in the Hanling et al. RCT, there was no improvement in depression, anxiety or pain in comparison to the sham group [48]. In two case reports, a left sided stellate ganglion block was found to increase anxiety symptoms, with improvement after an additional right sided block [56]. In a case series of 11 patients, it was noted that a SGB did not degrade neurocognitive performance in the form of reaction time, memory or in concentration at the expense of PTSD improvement [57].

### Pre-SGB PTSD Severity

While all the patients in the analyzed studies had a qualifying diagnosis of PTSD (minimum PCL score of 31–33), there were differences in the starting degree of PTSD severity, with some studies failing to note the starting severity all together. Olmsted et al. primarily enrolled patients with mild-moderate PTSD in their RCT showing a significant improvement after a SGB [49]. In the 166 patient case series by Mulvaney et al., the authors specifically separated their analysis by severity using a PCL score of 50 for a cutoff between higher severity and lower severity groups [45]. They found that both groups had a clinically significant improvement, however, the more severe group had a greater response, suggesting that the overall benefit of SGB may be greater in severe cases.

### Demographics and Trauma Types

PTSD has primarily been studied in the military population with trauma related to combat or military deployment. As the military make up is primarily male, this also results in most of the studies reflecting such a demographic. The Lipov et al. retrospective cohort analysis specifically looked at differences between males, females, civilians and non-civilians [43]. The authors analyzed 327 patients, of which 97 were military men, 85 were civilian men, 13 were military women and 132 were civilian women. 81% of patients had a clinically significant decrease in PCL-M or PCL-C scores after SGB. Statistical analysis showed that a significantly greater change occurred in men with a military background than in civilian men. Likewise, women with a military background had a greater reduction than in civilians. Overall, the SGB was equally effective for men and women across all trauma types. Another retrospective cohort analysis of 99 patients by Verrills et al. specifically looked at the non-civilian patient population and involved ~ 50% women [58]. The findings supported that there was a significant change observed in both men and women equally. 44 patients reported a specific trauma type resulting in 22 different trauma etiologies which all showed improvement. Mulvaney et al. also stratified 75 patients by trauma type in a retrospective analysis study extending out to 6 months [44]. The authors found that public service and childhood abuse patients were most likely to have improvement at the 1-week mark post injection, overall patients and unspecified trauma had the best improvement at 4 weeks, life threatening patients at 12 weeks and sexual abuse patients at 6 months. However, the results were durable in all trauma types. These studies support that SGB is effective for both genders in both military and non-military populations, irrespective of trauma type.

### Technique Considerations

#### Laterality

Historically, the stellate ganglion block was performed at C6 on the right side as the right brain hemisphere has been postulated to be involved in the human stress response [36, 59]. It has been thought that a right-sided SGB is associated with higher sympathetic dominance, while a left-sided SGB links to higher parasympathetic tone. Although no trial exists where a right sided block is compared to a left sided one, in the past 5 years, researchers started investigating the effects of combined bilateral blocks. Most commonly, the left sided block would occur within 24 h of the right sided one as to avoid airway compromise if the bilateral recurrent laryngeal nerve were inadvertently blocked. In one retrospective analysis of 205 military patients that looked at left sided blocks after right sided blocks, 90% of patients were found to respond to an initial right sided block with a decrease in PTSD symptoms [60]. Out of the 10% that did not respond to the right sided block, 90% that received a left sided block after had a clinically and statistically significant improvement. Other studies also support the safety and efficacy of a bilateral block, however, the only one that showed a greater score reduction in bilateral blocks was in the context of anxiety, otherwise single sided blocks were not compared against bilateral ones [44, 54, 55, 61]. When other indications are considered, left sided SGBs are preferred for ventricular arrhythmias and both unilateral and bilateral approaches have been used with success in long COVID symptoms, implying the role of both stellate ganglions in sympathetic mediated pathologies [62, 63].

### Block Repetition

A few of the studies observed the effects of repeating a SGB outside the baseline window. Most notably, the Olmsted et al. RCT repeated the injection at the 2-week mark [49]. As the only RCT showing clinical benefit, these findings propose whether this increases the rate of success. In the Hanling et al. RCT, 11 out of 27 treatment patients received a second injection [48]. While still not significantly significant in comparison to the sham group, there was a bigger effect noted with the group with repeated injections. In Mulvaney et al. 166 patient case series, the group repeated an injection within 24 h if no Horner’s syndrome was observed [45]. In 24 of those patients, a SGB was repeated at the 3-month mark showing equal efficacy to the first injection. This proposes that Horner’s syndrome could be utilized as a marker of a successful block and that, if an injection’s efficacy lessens, a second one can be performed that is equally as effective as the first. However, it is valuable to note that in the pain literature, sympathetic mediated pain improvement did not show correlation with occurrence of Horner’s syndrome in ultrasound guided therapeutic SGBs [64]. Horner’s syndrome also does not always indicate complete sympatholysis.

### Two Level Block with Superior Cervical Ganglion Block

Mulvaney et al. evaluated the effectiveness of 2 level SGB (C4 and C6) compared to the conventional method of single level C 6 SGB in 147 patients. Mean change in PCL 5 scores for the SGB was 25.2 (20.40246–29.84997 CI 95%) (*p* < 0.001) (*N* = 103) and 2LSB was 31.78 (26.05481–37.49065 CI 95%) (*p* < 0.001) (*N* = 44). At 4 weeks, 2 levels of SGB group had greater improvement as above but it was not statistically significant. There were no adverse events or complications reported in either group [65].

Lipov et al. reported the results of retrospective study in which superior cervical sympathetic ganglion was blocked at the ventrolateral aspect of C3 vertebral body using fluoroscopic guidance in 327 patients over 4 years. Among them, 244 patients received the dual block of SGB followed by SCGB. Average decrease in PCL score for men was 28.59 and women 29.2. Patients with military background had greater change than civilians and all types of traumas had a difference in PCL scores [43].

The same author also published a case series to visualize SGB impact through neuroimaging. Four patients who had C4 and C6 SGB on right followed by left side within 2 days underwent SPECT scans and PCL scores were collected. They reported a clinical difference in both primary and secondary PTSD and an increase in prefrontal pole, thalamus and inferior orbital activity correlates with the improved symptomatology [54]. Expanding on neurocognitive effects in this context, Mulvaney et al. reported improvement in mild-moderate TBI symptoms in PTSD patients who underwent 2 level and bilateral SGB one day apart [56].

Given most of the studies evaluated the benefit of SGB up to a month, Mulvaney et al. published the outcomes of bilateral 2 level SGB at 6 months in a retrospective study which included 75 participants, and 72 participants (96%) reported improvement in 6 months. The mean (SD) PCL-5 score at baseline was 51.34 (16.53) for all participants, with an average (SD) decrease of 28.48 (18.73) points after six months [44].

### Imaging

Technique can also vary by whether ultrasound or fluoroscopy or both are used for the block. Ultrasound-guided procedures limit radiation exposure and provide better visualization of vascular and structures such as the lung pleura, while also allowing for real-time needle tracking. Fluoroscopy better visualizes bony structures and typically is shot non-continuously, although live fluoroscopy is available for critical moments such as assessing for vascular uptake. The use of contrast dye can help confirm spread. There were no differences in effects noted regardless of whether fluoroscopy or ultrasound were utilized and no studies specifically compared fluoroscopy to ultrasound, showing that success may be dependent on operator preference, skill, equipment availability and anatomical considerations in patients. SGB staining was looked at in a cadaveric study in which a single experienced practitioner performed ultrasound and fluoroscopic guided injections on 10 cadavers [66]. The laterality for each procedure was randomized. Even though there was no statistically significant difference, ultrasound resulted in successful staining in 9 of 10, while fluoroscopy 6 out of 10. New approaches such as ultrasound guided supraclavicular stellate ganglion blocks between the prevertebral fascia have been proposed to decrease complications and offer additional benefits [67]. Ultrasound guided proximal intercostal blocks have also been proposed to produce consistent cervicothoracic sympathetic chain blockade [68]. Varying approaches will have to be compared as more data as available.

### Injectate

Across all studies with positive effects, all used 6–9 mL of either 0.5% Ropivacaine or 0.5% Bupivacaine without corticosteroids. For those with an additional SCGB, another 1.5-2 cc of the same local anesthetic was used. One investigator added clonidine while the rest were all plain [43]. The only study that did not find a positive correlation between SGB and PTSD was the RCT that only used 5 mL of 0.5% Ropivacaine, perhaps suggesting that at least 6 mL should be used [48].

### Length of Effect

While the overwhelming amount of literature appears to have a positive correlation between SGB and PTSD, all the investigations have varying follow up points (ranging from 1 day to 24 weeks). Mulvaney et al. performed two larger retrospective analysis with both short term (4–8 weeks) and long-term (3–6 month) follow ups [44, 45]. In one study of 75 non-military and military patients, 96% of patients had significant improvement at the 1 week, 1 month, 3 month and 6 month follow ups, regardless of trauma type [44]. In the other case series of 166 military patients, follow ups occurred at 1 week, 4–8 weeks and 12–24 weeks, with 70% of patients showing clinically significant improvement persisting beyond 3–6 months, regardless of PTSD severity [45]. These studies argue that the results are durable.

### Adjunct Therapies

One variable that is tough to assess across studies adjunct therapies prior and during the study period. Most of the patients included in the investigated literature had already failed standard therapy or had received some care prior. Positive effects seem to be observed in both patients that were therapy naïve and treated patients. For example, a handful of smaller case series looked at individuals treated for at least a year with a SSRI and psychotherapy while another looked at patients that had not received any treatment within 6 months and both found statistically significant positive correlations in most patients [47, 53, 57]. Some studies purposely looked at SGB in conjunction with therapy. For example, a 12 military patient nonrandomized clinical trial showed that SGB, done in conjunction with prolonged exposure therapy sessions, provided a clinically significant reduction in PTSD symptoms in 88.9% of patients and 87.5% no longer met criteria for PTSD [69]. In another 14 military patient nonrandomized clinical trial, SGB done in conjunction with weekly psychotherapy exercises also found a statistically significant improvement [70].

### Satisfaction and Safety of SGB

There is overall success and acceptance of SGB treatment among service members. A pilot program conducted with combat veterans demonstrated that the SGB treatment exhibited a high rate and level of response [51, 71]. Furthermore, the program evaluation analysis indicated that the therapy reduced stigma and was well-accepted by service members. In a satisfaction survey with 110 respondents from 250 SGB, 100% of patients were overall satisfied and would recommend it to other patients [72]. 95% of these patients would undergo another and no complications were reported.

SGBs are generally well-tolerated and considered low risk. Most adverse events are mild and transient, including injection discomfort, minor bruising, temporary Horner’s syndrome or voice changes [73]. More serious complications, such as pneumothorax, hematoma and local anesthetic systemic toxicity, occur in less than 1–2% of cases. Image guidance continues to improve the safety profile. Dual-level blocks appear to be safe, but larger scale comparisons are needed to see if there is elevated risk between single and double injections.

#### Challenges and Limitations

While the highest quality evidence in RCTs appears to be mixed, much of the current literature supports the efficacy of SGBs in the treatment of PTSD as evidenced by this review. Further large, randomized, clinical trials with clear designs and protocols must be performed to assess the efficacy of the treatment in non-biased and generalizable study populations. There are four active randomized controlled trials that are ongoing in clinicaltrials.gov that may provide further data [74–77]. The combination of these will offer more information on treatment versus control groups for SGB injections, compare the effectiveness of therapy in conjunction with injections, and provide fMRI comparisons. Although SGBs have been supported to be effective across trauma types, further insight can be made into trauma and non-trauma subtypes. For example, in the military population, active-duty vs. retired, deployment sites, extent of involvements and additional injuries that were sustained may play a role. Some of the blocks utilized sedation, which might alter the results. Patients may also have secondary gains such as graduating their treatment programs, compensation for studies or applying for disability.

Most of the studies included were retrospective cohort studies and uncontrolled case series, which are inherently subject to limitations. These studies risk selection bias as patient inclusion is often determined by referral patterns and treatment availability and may capture patients more likely to respond to interventions. Some of the included studies originated from the same investigative groups, potentially introducing overlapping patient populations, limiting external validity and reducing generalizability. It may also create observer bias if the investigator anticipates positive results from previous assessments. Without randomized controlled groups, it is challenging to distinguish true treatment effects from placebo response, regression to the mean, or the natural disease progression and symptom fluctuation in PTSD. While most studies incorporated standardized outcome measures, there were variable follow-up durations for comparability across these studies, which do not account for attrition. Confounders such as PTSD severity, comorbid psychiatric conditions and concurrent pharmacologic or psychotherapeutic interventions, may also limit the results.

## Conclusion

SGBs have emerged as a potential intervention for PTSD, with much of the existing literature suggesting benefit across multiple symptom domains. The strongest effects have been observed in hyperarousal, though improvements have also been reported in re-experiencing and avoidance. Evidence indicates that outcomes may be more pronounced in patients with greater baseline severity. Despite these findings, the current literature is limited by small sample sizes, heterogenous methodologies, and a lack of long-term follow up. Larger, well-designed randomized controlled trials are required to establish the efficacy, durability and appropriate clinical role of SGB in the treatment of PTSD. SGBs should be considered in conjunction with other PTSD treatment.

## Key References

- Lipov EG, Jacobs R, Springer S, blet al. Utility of Cervical Sympathetic Block in Treating Post-Traumatic Stress Disorder in Multiple Cohorts: A Retrospective Analysis. Pain Physician. 2022 Jan;25(1):77-85.
  - ○ This is a large retrospective cohort analysis that supports significant reductions in PTSD symptoms after SGB across diverse trauma types and both military and civilian patients. It is also the largest study to include a superior cervical ganglion block and show it is safe and effective.
- Mulvaney, S. W., Mahadevan, S., Dineen, K. J., et al. (2025). Long-Term Durability of Bilateral Two-Level Stellate Ganglion Blocks in Posttraumatic Stress Disorder: A Six-Month Retrospective Analysis. Clinical and Translational Neuroscience, 9(1), 7. Doi: 10.3390/ctn9010007
  - ○ A retrospective analysis which investigated bilateral, 2-level SGB, stratified by trauma type, to show that public service and childhood abuse patients were most likely to have improvement in PTSD symptoms at 1 week, overall patients and unspecified trauma at 1 month, life threatening patients at 3 months and sexual abuse trauma at 6 months. This study is unique in that it follows patients up to 6 months and showed that the results are durable at this time point.
- Blakey SM, Rae d KL, Hirsch S, et al. Differential posttraumatic stress disorder symptom cluster response to stellate ganglion block: secondary analysis of a randomized controlled trial. Transl Psychiatry. 2024 May 29;14(1):223. doi: 10.1038/s41398-024-02926-8. Erratum in: Transl Psychiatry. 2024 Jun 25;14(1):265. doi: 10.1038/s41398-024-02983-z.
  - ○ This is a secondary analysis of a randomized controlled trial that specifically served as a descriptive analysis to see what symptoms may be most improved from a SGB. Hyperarousal, reactivity, and re-experiencing symptoms appear to be most improved in clinician ratings.
- Verrills, Paul & Lipov, Eugene & Wright, Robert & Sethi, Zubin. (2023). Efficacy of Right-Sided Cervical Sympathetic Block in Treating Post-Traumatic Stress Disorder in Australian Cohorts: A Retrospective Analysis. Research and Practice in Anesthesiology – Open Journal. 7. 15-20. 10.17140/RPAOJ-7-136
  - ○ A retrospective cohort analysis showing that a 2-level SGB is effective in both military men and women across 22 different trauma types.

## Data Availability

No datasets were generated or analysed during the current study.
